# Management of Anticoagulation in Athletes

**DOI:** 10.3390/jcm15187213

**Published:** 2026-09-17

**Authors:** Pranavi Bavishi, Prashant Raghavendran

**Affiliations:** 1 Department of Hematology Oncology, Northwestern Medicine, Chicago, IL 60611, USA; pranavi.bavishi@northwestern.edu; 2 Department of Pediatrics, Division of Hematology/Oncology, Emory University School of Medicine, Atlanta, GA 30322, USA

**Keywords:** anticoagulation, thrombosis, athletes

## Abstract

Venous thromboembolism (VTE) in athletes presents a unique clinical challenge that contradicts the traditional perception of athletes as low-risk individuals. While regular exercise mitigates cardiovascular risk and improves venous flow through frequent mobilization, injuries, inappropriate mechanics of participation, comorbid medical conditions, recreational utilization of performance-enhancing medication, and underlying thrombophilia all create unique circumstances for thrombosis in this patient population. Individual consideration of anticoagulant agent use, duration of treatment, and pauses in treatment depend on underlying bleeding and thrombosis risk and level of activity and lifestyle for each patient. Continuation of anticoagulation after return to participation in sports also requires thorough risk–benefit assessment factoring in overall health, and hinges partially on the ability to reverse the effect of anticoagulation in dire situations. Guidance about continuation of a particular sport if long-term anticoagulation is needed depends on shared decision-making about morbidity and quality of life. Adjunct methods to prevent further thrombosis and manage post-thrombotic syndrome are also important for athletic longevity. There is a growing body of literature regarding evaluation and management of athletes with thrombosis, and the authors aim to summarize recommendations as they apply to pediatric and adult athletes. However, it is noted that continued study of this topic is needed to create more generalized recommendations rather than considerations for individual patient management, as discussed within this article.

## 1. Introduction

Venous thromboembolism (VTE) in athletes presents a unique clinical challenge that contradicts the traditional perception of athletes as low-risk individuals. While moderate exercise confers cardiovascular protection, competitive athletes face distinct thrombotic risk factors that necessitate specialized management strategies, balancing anticoagulation with bleeding risk and athletic performance.

High-intensity training, repetitive mechanical stress, dehydration, travel-related immobility, and sport-specific biomechanical factors may all contribute to thrombogenesis in otherwise healthy individuals [1]. These factors are in addition to known thrombotic predisposing factors in the general population, including anatomic anomalies and thrombophilia.

Anticoagulation therapy, while essential for preventing recurrence and reducing morbidity, carries implications for training modification, return-to-play decisions, and overall athletic career trajectory, which can have a profound impact on an athlete’s quality of life.

## 2. Epidemiology of Thrombotic Conditions in Athletes

Although athletes are generally considered healthy, approximately 1 in 1000 athletes will experience a post-exercise thrombotic event [1]. Overall, the incidence of VTE in athletes is lower than the general population; however, there are some sport-specific factors that raise the thrombotic risk in some patients [2].

Athletes, like other individuals, experience thrombotic risk through a disruption of Virchow’s triad—hypercoagulability, venous stasis, and vessel wall injury [2]. However, the mechanism of disruption differs in this specialized population. Hypercoagulability in athletes results from hemoconcentration induced by exertion, dehydration, and polycythemia [2]. Venous stasis occurs during prolonged immobilization from long-distance travel for competition, post-injury recovery, and overdevelopment of musculature. Vessel wall injury, the most important contributor to thrombosis in this group, results from repetitive microtrauma to venous and arterial walls from sport-dependent trauma, high metabolic rates, and repetitive blood monitoring [1].

Additional risk factors include intense training, trauma, immobilization from injuries, oral contraceptive use in female athletes, and frequent long-haul flights [3]. In female athletes, oral contraceptives (especially within the first six months of administration) represent a particularly significant risk factor, present in 73.7% of young women diagnosed with exercise-associated VTE [3].

## 3. Thrombotic Risk Assessment in Athletes

### 3.1. Incidence and Unique Predisposing Factors

Etiology of VTE in athletes vary by type of sport. One study looking at 154 cases of thrombosis in association with athletic activity found that 89 patients had upper extremity deep vein thrombosis (DVT), 53 had lower extremity DVT, and 12 had pulmonary embolism. The sports that were more associated with an upper extremity DVT were baseball and weightlifting [2].

Further, 38% of upper extremity DVT cases were associated with venous thoracic outlet syndrome (VTOS). VTOS results from compression of the subclavian vein within the costoclavicular space, leading to episodic venous outlet obstruction with edema and functional symptoms [4]. Sports involving repetitive overhead arm movements (particularly baseball, volleyball, swimming, and wrestling) predispose to VTOS through cumulative compression injury [4].

Lower extremity DVT predominates in endurance sports and ball sports where prolonged standing, running, and lower extremity trauma occur.

Pulmonary embolism (PE) is mainly derived from propagation of other VTE from which portions fracture and travel to the pulmonary vasculature [5]. Congenital cardiac disease that contributes to stasis of blood flow portends additional risk, as does blunt trauma to the torso sustained during sports-related contact that causes local inflammation.

#### 3.1.1. Atrial Fibrillation in Athletes

High-volume endurance training (>45 metabolic-equivalent-hours per week) is associated with increased atrial fibrillation (AF) prevalence [6]. The relationship between exercise volume and AF follows a U-shaped curve, with both sedentary behavior and extreme endurance training increasing risk [6]. In a study of 208,654 Swedish skiers with a mean age of 37, 1.2% developed atrial fibrillation in the eight-year follow up of the study, and the incidence was highest in athletes with the fastest finishing times and those who had completed the greatest number of races. Male skiers had a higher incidence compared to female skiers. Older age, male sex, and endurance training are associated with an increased incidence of atrial fibrillation [7].

#### 3.1.2. Performance-Enhancing Therapies and Thrombosis Risk

Though inadmissible in a majority of amateur and professional sports, performance-enhancing agents, such as corticosteroids and erythropoietin-stimulating agents, can confer significant thrombosis risk. Glucocorticoids can cause polycythemia and increased platelet aggregation as well as decreased endothelial integrity and thus vascular injury, while erythropoietin-stimulating agents can cause hyperviscosity and thus blood stasis via promoting erythrocytosis [8,9]. Many other agents, when used in this context, have anecdotal associations with thrombosis that are compounded with other patient risk factors and warrant further exploration [9]. Inquiry about use of high-risk pharmaceuticals should always be considered in athletes with thrombosis.

#### 3.1.3. Thrombophilia

##### Inherited Thrombophilia

Hereditary thrombophilia (Factor V Leiden mutation, prothrombin gene mutation, protein C deficiency, protein S deficiency, and antithrombin deficiency) or family history of VTE was identified in 16% of upper extremity DVT cases and 30% of lower extremity DVT cases in athletes [2]. When acquired risk factors compound upon genetic predispositions to hypercoagulability, overall VTE risk substantially increases, particularly for lower extremity DVT [1].

##### Acquired Thrombophilia

Athletes can also be prone to developing antiphospholipid antibody syndrome, which can predispose to arterial or venous thrombosis. This can be primary or secondary to other autoimmune conditions and may necessitate long-term anticoagulation if markers of elevated antiphospholipid antibodies persist according to the diagnostic criteria. Primary prevention can be performed with antiplatelet therapy in selected high-risk antiphospholipid antibody profiles, while thrombotic antiphospholipid syndrome generally requires long-term anticoagulation [10].

## 4. Management

### 4.1. Acute Treatment

Anticoagulation should be initiated promptly when DVT/PE is diagnosed or if high clinical suspicion exists [11]. The acute management phase encompassing the first 5 to 21 days aims to halt active thrombus propagation [12]. Options for initial management include immediate treatment with direct oral anticoagulants (DOACs; rivaroxaban 15 mg twice daily for 21 days or apixaban 10 mg twice daily for 7 days), parenteral anticoagulation followed by a DOAC, or parenteral anticoagulation overlapped with warfarin for at least 5 days until INR is therapeutic between a goal of 2.0 and 3.0 [11,12].

DOACs are the preferred choice for most athletes with DVT/PE due to their favorable efficacy and safety profile [11]. Evidence-based guidelines favor outpatient treatment with a DOAC-based regimen in most patients with adequate home support and manageable symptoms [11]. For athletes requiring rapid reversibility, such as those potentially undergoing catheter-directed therapy, individuals in need of multiple surgical treatments, or those who are critically ill, unfractionated heparin may be preferred in the immediate short term, followed by low-molecular-weight heparins (LMWHs), such as enoxaparin, due to ease of monitoring and shorter half-life [11,12]. Vitamin K antagonists are not preferred due to dietary restrictions and difficulty in maintaining INR in the therapeutic range.

Patients with isolated distal DVT may require less extensive treatment. However, athletes with risk factors for progression—including extensive thrombosis (>5 cm lengths), symptoms from clot burden, proximity to proximal veins, or previous VTE history—should receive longer courses of treatment if risks of therapy are not significant [12].

### 4.2. Duration of Anticoagulation

Patients with acute DVT/PE generally receive three to six months of anticoagulation during the primary treatment phase [12]. Duration beyond three to six months depends on whether the DVT/PE was provoked or unprovoked, bleeding risk, and individual athlete factors [11,12].

Athletes with DVT/PE provoked by major transient risk factors (surgery, trauma, and prolonged immobilization) should receive 3–6 months of anticoagulation, after which treatment can be discontinued due to low recurrence risk [11,12]. Those with unprovoked DVT or persistent risk factors face higher recurrence rates (10% at 1 year and 36% at 10 years after discontinuation) and should be considered for extended or indefinite anticoagulation [11,12].

The decision to continue anticoagulation beyond three to six months requires balancing recurrence risk against bleeding risk [12,13]. Major bleeding occurs at approximately 1–2% annually with extended anticoagulation and is nearly three times more likely to be fatal than recurrent VTE (11% vs. 4%) [12]. Though this is a relatively small percentage, frequent discussions should occur with patients about bleeding symptoms prior to initiating and during the course of anticoagulation. Athletes with high bleeding risk—defined by advanced age (>65 years), needing antiplatelet therapy, chronic renal impairment, anemia, or bleeding history—should be considered for reduced anticoagulation dosing if thrombosis risk outweighs bleeding risk. It must be noted that chronic anemia, whether due to iron deficiency or inflammation, as well as chronic renal impairment may pose added thrombosis risk as well. Weighing thrombosis risk factors versus bleeding risk factors for each patient with such conditions is important prior to long-term decision-making. Furthermore, patients with a significant bleeding phenotype could be considered for anticoagulation cessation after the initial treatment course if prolonged therapy is of excess harm [12,13].

### 4.3. VTOS Management

Acute upper extremity DVT from VTOS (also known as Paget–Schroetter syndrome) requires multimodal treatment. Initial management includes anticoagulation with catheter-directed thrombolysis for extensive swelling or functional impairment [14,15]. Definitive treatment involves surgical decompression via paraclavicular thoracic outlet decompression with first rib resection, anterior scalenectomy, and venolysis, typically performed 1–3 months after initial diagnosis [15,16]. Athletes who underwent catheter-directed thrombolysis at initial diagnosis were 32% less likely to need vein reconstruction at surgery and 60% less likely to have arm swelling at follow-up [17]. Most patients can discontinue anticoagulation and return to high-level athletic activity after surgical decompression [18].

### 4.4. Return-to-Play Considerations

Physical exercise after DVT is safe, improves quality of life, reduces pain, and decreases post-thrombotic syndrome severity [19]. Early mobilization in the acute phase improves quality of life and pain, with significant reduction in post-thrombotic syndrome severity at two years after thrombosis identification [19]. Prolonged supervised exercise programs and physical therapy when indicated result in improved quality of life and positive effects on venous insufficiency symptoms and muscle function [19].

Return to competitive sports while taking anticoagulation depends on sport classification and bleeding risk. Athletes in low-risk sports may return to play during anticoagulation, while those in collision or high-impact sports require individualized shared decision-making considering career aspirations, medical safety, and willingness to accept bleeding risk [13]. For athletes with provoked DVT from transient risk factors, return to full competition may be feasible after completing the three-month anticoagulation course, while those requiring extended anticoagulation face ongoing participation restrictions in high-risk sports [13,20].

### 4.5. Challenges of Balancing Thromboprophylaxis with Bleeding Risk and Athletic Performance

Anticoagulation in athletes requires navigating competing priorities: preventing thromboembolic complications while minimizing bleeding risk during high-impact activities and maintaining competitive performance. Traditional anticoagulation guidelines, developed for sedentary populations, inadequately address the unique demands of athletic competition. The inability to rapidly reverse anticoagulation during trauma or injury, coupled with the unpredictable nature of sports injuries, creates management complexity [13].

### 4.6. Anticoagulation Options and Pharmacologic Considerations

#### 4.6.1. Direct Oral Anticoagulants (DOACs)

##### Preferred Agents

DOACs are generally preferred for athletes due to their favorable efficacy and safety profile compared to vitamin K antagonists [13]. In meta-analyses, DOACs demonstrated noninferiority to warfarin for recurrent VTE (2.0% vs. 2.2% for warfarin) with significantly lower major bleeding rates (1.1% vs. 1.8%), intracranial hemorrhage (0.1% vs. 0.3%), and fatal bleeding (0.1% vs. 0.2%) [21].

##### Pharmacokinetic/Pharmacodynamic Properties

The four available DOACs—rivaroxaban, apixaban, edoxaban, and dabigatran—differ in pharmacokinetic profiles relevant to athletic scheduling [22]. Rivaroxaban and apixaban can be initiated immediately without parenteral lead-in, while dabigatran and edoxaban require 5–10 days of heparin or low-molecular-weight heparin (LMWH) before oral therapy [11]. Half-lives range from 5–9 h (rivaroxaban) to 12–17 h (dabigatran and apixaban) [22]. Rivaroxaban demonstrates 66% renal excretion, apixaban 27% renal with 73% biliary/intestinal, edoxaban 50% renal with 50% hepatic/biliary, and dabigatran 80% renal [22]. It must be noted that the half-lives of most DOACs are longer than those of LMWH and unfractionated heparin, so providers must be wary of their use in individuals who have an increased bleeding risk due to inherited hemostasis disorders, have an increased bleeding phenotype, or have a transient increase in bleeding risk due to critical illness, trauma, or repetitive surgery.

##### Limitations

Rivaroxaban is not recommended for use in patients with a CrCl (creatinine clearance) of less than 15 mL/min and in those with Childs Pugh Class B–C liver disease. Eliquis is not recommended for use in patients with Childs Pugh Class C liver disease and has not been studied in patients with a CrCl of less than 25 mL/min. Edoxaban is not recommended in Childs Pugh Class B–C liver disease and also in patients with a CrCl less than 15 mL/min. Dabigatran is not recommended for use in patients with a CrCl of less than 30 mL/min [22].

#### 4.6.2. Vitamin K Antagonists (Warfarin)

##### Limitations in Athletic Populations

Warfarin presents significant challenges for athletes. Diet changes around competitions alter vitamin K intake, causing INR fluctuations and unpredictable anticoagulation levels [13]. The long half-life (20–60 h) and variable metabolism through CYP2C9, CYP1A2, CYP2C19, and CYP3A4 create management complexity [22].

##### Strong Discouragement for Contact or High-Impact Sports

Athletes requiring warfarin should be strongly discouraged from contact or high-impact sports participation due to the inability to safely manage bleeding and thrombotic risks [13]. The unpredictability of anticoagulation levels coupled with trauma creates the potential for significant bleeding risk in collision sports [13].

#### 4.6.3. Low-Molecular-Weight Heparin (LMWH)

##### Role as Alternative Therapy

LMWH serves as an alternative when DOACs are contraindicated [5,20]. LMWH offers advantages, including subcutaneous administration, predictable anticoagulation when weight-based dosing is used, and a lower HIT risk than unfractionated heparin [5].

##### Practical Considerations for Athletes

LMWH requires once- or twice-daily subcutaneous injections, which may be logistically challenging during athletic events [5]. However, predictable pharmacokinetics and the ability to time doses around athletic events provide flexibility [20]. In pediatric populations, prolonged LMWH use (at least 12 months of consistent use) raises concerns about bone mineralization, particularly relevant for young athletes [23].

#### 4.6.4. Antiplatelet Therapy

##### Dual Antiplatelet Therapy Considerations

Dual antiplatelet therapy (DAPT) increases bleeding risk compared to monotherapy, particularly in sports with collision or impact potential [13,24]. Athletes receiving antiplatelet therapy should undergo individualized risk assessment, with particular attention to collision and impact sports [13,24].

When patients on DAPT are undergoing a procedure or surgery, risk stratification must be used to determine whether temporary discontinuation of DAPT will be tolerated. If a patient does not have a high risk of a cardiac event, it is okay to hold aspirin for 7–10 days prior to surgery and it can be resumed typically 24 h after surgery, when adequate hemostasis has been obtained. For patients with a high risk of a cardiac event, such as those who had a myocardial infarction in the past three months, patients can continue taking aspirin but could hold clopidogrel. In patients who have had a bare metal stent placed in the past six weeks, surgery should be postponed if possible, or if surgery must be done, DAPT should be continued, as these patients are at high risk of stent thrombosis [24].

#### 4.6.5. Reversibility of Anticoagulation Agents

The reversibility of anticoagulation is an important consideration in athletes due to the risk of traumatic injury, urgent surgery, and bleeding complications during competition. DOACs have shorter half-lives than warfarin and now have specific reversal agents available, improving safety in emergency situations [5,13].

Idarucizumab is a monoclonal antibody fragment approved for reversal of dabigatran and produces rapid neutralization of anticoagulant activity within minutes. Andexanet alfa acts as a decoy factor Xa protein and reverses factor Xa inhibitors, including rivaroxaban and apixaban. However, this medication has been removed from the United States market due to the risk of thrombosis outweighing the potential benefit in anticoagulation reversal [25]. In settings where medication-specific agents are unavailable, four-factor prothrombin complex concentrate (4F-PCC) may be used for reversal of factor Xa inhibitors when specific reversal agents are unavailable or unsuitable [3,26,27].

Warfarin reversal requires vitamin K administration and, in severe bleeding, supplementation with fresh frozen plasma or 4F-PCC [3]. Unfractionated heparin can be rapidly reversed with protamine sulfate, while low-molecular-weight heparins are only partially reversible with protamine [3].

For athletes participating in contact or collision sports, the ability to rapidly reverse anticoagulation may influence anticoagulant selection and return-to-play discussions. Shared decision-making should incorporate medication half-life, access to reversal agents, proximity to emergency medical care, and sport-specific bleeding risk [13].

### 4.7. Sport-Specific Considerations

#### 4.7.1. Individualized Treatment Approaches

##### Risk–Benefit Assessment

Each athlete requires individualized assessment weighing thromboembolic risk, hemorrhagic risk, sport-specific trauma potential, competitive aspirations, and personal values [13]. Factors include whether the VTE was provoked or unprovoked, presence of persistent risk factors (cancer and thrombophilia), bleeding risk scores, and sport classification (Table 1) [13].

##### Intermittent Dosing Regimens with DOACs

Tailored anticoagulation plans leveraging DOAC pharmacokinetics permit timing drug administration around competitive events. With newer assays allowing the ability to monitor levels of DOACs as well as other anticoagulants, pharmacokinetic testing can be performed in athletes to devise an intermittent dosing plan after the initial treatment phase for secondary prophylaxis to attempt to balance thrombosis and bleeding risks [13,28].

We present the case of a patient who is benefitting from intermittent dosing of rivaroxaban (Table 2).

This patient had pharmacokinetic studies done to evaluate peak and trough levels on rivaroxaban. Though they were healthy with normal renal and hepatic function, the levels were felt to be helpful in determining the ability to hold and restart anticoagulation at apportioned times around varying intensities of activity without predisposing to increased bleeding or thrombotic risk. The care team was able determine a plan for him so that he can continue to play soccer competitively—for soccer matches, he holds his rivaroxaban the evening before and the evening of his game and resumes the day after his match if no trauma or bleeding issues occur. For soccer practice, he takes a half dose of rivaroxaban the day before the game and resumes a 20 mg dose the evening of practice, if there was no trauma or bleeding. This patient has been following an intermittent dosing regimen and has been able to successfully participate in competitive higher-risk sports while on lifelong rivaroxaban.

However, this approach lacks robust safety data and should be pursued only with comprehensive shared decision-making [28]. This process must be individualized and requires understanding from the athlete that any complications in such a process may require modifying participation in sports.

##### Shared Decision-Making: Essential Components

Athlete–physician discussions must address career aspirations, medical safety, thromboembolic risk, bleeding risk, sport-specific trauma potential, and anticoagulation options [13,20]. Optimal care requires collaboration among cardiologists, hematologists, sports medicine specialists, athletic trainers, interventional radiologists, and vascular surgeons. Cardiologists and hematologists provide anticoagulation expertise, sports medicine specialists understand sport-specific demands, athletic trainers monitor day-to-day symptoms, and vascular specialists manage complications like post-thrombotic syndrome [13].

#### 4.7.2. Managing Post-Thrombotic Syndrome

Post-thrombotic syndrome management includes compression stockings, though their impact on athletic performance requires consideration [15]. Compression therapy may affect proprioception, comfort, and performance in certain sports. Multidisciplinary teams can optimize compression strategies, balancing symptom control with athletic function. In more severe post-thrombotic syndrome (severity indicated by the Villalta score), intervention to attempt revascularization may be considered as well, but this may involve longer periods of anticoagulation or antiplatelet therapy and longer periods of recovery before return to play, so benefits and risks must be discussed with athletes based on their symptoms. Post-PE syndrome leads to prolonged dyspnea at rest and on exertion after management and improvement of thrombosis that can limit exercise tolerance in athletes [29]. There is a role for serial monitoring of cardiac and pulmonary function as well as pulmonary rehabilitation programs to help limit the effects of this condition and allow athletes to resume sports participation at a competitive level, however the degree of vascular involvement of initial thrombosis, as well as resultant surrounding tissue infarction, can be barriers to recovery.

#### 4.7.3. Periodic Reassessment

##### Thromboembolic Risk Re-Evaluation

Regular reassessment of VTE recurrence risk considers resolution of provoking factors, development of new risk factors, and duration of anticoagulation. For provoked VTE with major transient risk factors, anticoagulation typically stops at three months. Unprovoked VTE or persistent risk factors warrant extended or indefinite anticoagulation [12].

Ongoing bleeding risk assessment includes accounting for new medications (NSAIDs and antiplatelet agents), changes in renal or hepatic function, and bleeding events [13]. Sport participation changes (transitioning from low-risk to high-risk sports) necessitate anticoagulation strategy revision.

Treatment modifications respond to changing risk profiles, competitive schedules, and athlete preferences [13]. Athletes transitioning from competitive to recreational participation may accept different risk–benefit trade-offs.

### 4.8. Special Considerations in Pediatric Patients

#### 4.8.1. Drug Metabolism Differences

Pediatric patients demonstrate distinct pharmacokinetic profiles compared to adults [23,30]. The DOACs rivaroxaban and dabigatran are licensed for pediatric VTE treatment, providing convenient alternatives to standard-of-care anticoagulants [30]. However, cumulative experience using DOACs in pediatrics, particularly for those with coexisting chronic illnesses, remains sparse [30].

#### 4.8.2. Enoxaparin and Bone Mineralization

Prolonged LMWH use in pediatric patients raises concerns about bone mineralization [23]. This consideration is particularly relevant for young athletes requiring extended anticoagulation, as bone health directly impacts athletic performance and long-term skeletal development.

#### 4.8.3. Compliance Challenges

Medication adherence in pediatric and adolescent athletes presents unique challenges [30]. Subcutaneous LMWH injections may be particularly difficult for young athletes to maintain during school, training, and competition. DOACs offer improved convenience through oral administration and reduced monitoring requirements [30].

#### 4.8.4. Incidence of Thrombophilia, Duration of Treatment, and Sports Participation Decisions

More than 90% of pediatric VTE events are provoked [23]. The Kids-DOTT trial demonstrated that 6 weeks of anticoagulation was noninferior to 3 months for provoked VTE in patients younger than 21 years [29]. These findings inform return-to-play decisions, as a shorter anticoagulation duration may facilitate earlier sports resumption. However, inherited thrombophilia requires individualized assessment regarding long-term sports participation safety.

## 5. Future Directions

### 5.1. Need for Validated Treatment Regimens

Current anticoagulation management in athletes relies heavily on expert opinion and extrapolation from non-athletic populations [13,20]. Prospective studies evaluating the safety and efficacy of sport-specific anticoagulation protocols are needed. Validated regimens should address optimal DOAC selection, dosing strategies, and return-to-play criteria across different sports.

### 5.2. Research Gaps in Intermittent Anticoagulation Safety

While intermittent DOAC dosing around competitive events represents an attractive strategy, robust safety data are lacking [28]. Future research should evaluate thromboembolic and bleeding outcomes with various intermittent dosing protocols, identify athletes most suitable for this approach, and establish monitoring parameters to ensure safety.

### 5.3. Development of Sport-Specific Guidelines

Current guidelines inadequately address sport-specific considerations [13]. Future guideline development should incorporate sport classification systems, trauma risk stratification, and evidence-based recommendations for each sport category. Guidelines should also address unique scenarios like international travel for athletic events, altitude training, and extreme environmental conditions.

## 6. Conclusions

Anticoagulation management in athletes requires balancing effective thromboprophylaxis with bleeding risk and athletic performance. DOACs offer advantages over warfarin through more predictable pharmacokinetics enabling individualized dosing strategies. Shared decision-making, multidisciplinary collaboration, and periodic reassessment optimize outcomes. Future research must establish validated treatment regimens, evaluate intermittent anticoagulation safety, and develop sport-specific guidelines to better serve this unique population.

## Figures and Tables

**Table 1:** Risk classification of sports for athletes on anticoagulation therapy management strategies.

Risk Category	Example Sports	Recommendation
***Non-contact/low-collision sports***		
**Low Risk**	• Swimming and water aerobics • Walking, hiking (non-extreme) • Golf • Yoga, Pilates • Table tennis • Archery • Recreational cycling (flat terrain) • Dancing (non-contact) • Rowing/kayaking (calm water)	Generally permitted. Minimal trauma risk; appropriate protective equipment advised.
***Limited-contact/moderate-collision sports***		
**Moderate Risk**	• Tennis (singles and doubles) • Badminton • Volleyball (recreational) • Baseball/softball • Track and field • Gymnastics • Skiing • Road cycling • Equestrian (recreational riding) • Martial arts (non-sparring)	Use with caution. Individualized case-by-case assessment; protective equipment essential.
***Contact/high-collision sports***		
**High Risk**	• American football/rugby • Ice hockey • Boxing (sparring/competition) • Wrestling/judo • Downhill mountain biking • Alpine ski racing • Motocross/motorsports • Lacrosse (contact) • Basketball (competitive/contact) • Soccer (competitive)	Generally discouraged. High risk of significant traumatic bleeding; substantial risk of intracranial hemorrhage. Requires thorough individualized risk–benefit discussion.
***Special Considerations: Extreme/high-altitude/underwater sports***		
**Variable/High Risk**	• Scuba diving • Skydiving/parachuting • Rock climbing/mountaineering • White-water rafting/kayaking • Bungee jumping	Individualize carefully. Remote trauma risk, limited emergency access, and physiologic stressors compound bleeding hazard.

**Table 2:** Timeline of thromboembolism and treatment in a 15-year-old patient.

	9/5/23	11/30/23	3/17/25	6/13/25
**Event**	**VTE #1—popliteal DVT** 15 M presents with L calf swelling and pain, found to have L popliteal vein thrombus. Found to have antithrombin III (ATIII) deficiency (52%), family hx of ATIII deficiency.	**Follow-up imaging** Popliteal thrombus resolved. No new symptoms. Tolerating rivaroxaban well. Dose stepped down.	**VTE #2—calf DVT** Presents with new L leg pain. Calf DVT confirmed. Provoked in the setting of a calf injury + ATIII deficiency.	**Follow-up—VTE #2** US (2/2) confirms L calf DVT resolved. Decision: indefinite anticoagulation.
**Intervention**	**Enoxaparin → rivaroxaban** Started on enoxaparin on admission, transitioned to rivaroxaban therapeutic dose prior to discharge.	**Rivaroxaban 10 mg/day** Stepped down to prophylactic dosing.	**Rivaroxaban increased** Back to therapeutic dosing of 20 mg daily.	**Rivaroxaban indefinitely** Therapeutic dosing ongoing with intermittent dosing prior to soccer matches, practices, and tournaments.
**Imaging**	**Doppler venous US** Popliteal thrombus confirmed.	**Doppler venous US** Thrombus resolved.	**Ultrasound** Calf DVT confirmed.	**Ultrasound** DVT resolved.

## Data Availability

No new data were created or analyzed in this study. Data sharing is not applicable to this article.
